# Applicability of health promoting lifestyle profile-II for postmenopausal women in Sri Lanka; a validation study

**DOI:** 10.1186/s12955-020-01371-7

**Published:** 2020-05-06

**Authors:** Nirmala Rathnayake, Gayani Alwis, Janaka Lenora, Sarath Lekamwasam

**Affiliations:** 1 Department of Nursing, Faculty of Allied Health Sciences, University of Ruhuna, Galle, Sri Lanka; 2 Department of Anatomy, Faculty of Medicine, University of Ruhuna, Galle, Sri Lanka; 3 Department of Physiology, Faculty of Medicine, University of Ruhuna, Galle, Sri Lanka; 4 Population Health Research Centre, Department of Medicine, Faculty of Medicine, University of Ruhuna, Galle, Sri Lanka

**Keywords:** Cross cultural adaptation, Health promoting lifestyle profile-II, Psychometric properties, Postmenopausal women, Reliability, Sinhala version, Validity

## Abstract

**Background and objective:**

Health Promoting Lifestyle Profile-II (HPLP-II), developed in the West, evaluates 52 health promoting behaviors (HPB) under six subscales. In this study we evaluated the applicability of HPLP-II to assess the HPB of postmenopausal women (PMW) conversant in the Sinhala language in Sri Lanka.

**Methods:**

The Sinhala version of HPLP-II was adapted following standard methodology of cross cultural adaptation. It included forward and backward translations, review by an expert group, focus group discussion and pre-testing. It was self-administered among randomly selected healthy, Sinhalese, community-dwelling PMW (*n* = 245, aged 55.9 ± 3.4 years), along with the Short Form 36 (SF-36) survey. The Sinhala version of HPLP-II was re-administered among a subsample (*n* = 105) after two weeks of first administration. Psychometric properties - reliability and validity, were evaluated.

**Results:**

In the Sinhala version of HPLP-II, both internal consistency (Cronbach’s alpha = 0.98) and test-retest reliability (intra class correlation / ICC = 0.98, 95%CI = 0.97–0.99) were high. Structural validity assessment with Factor analysis using Principal Component Analysis extracted seven factors explaining 80.65% cumulative variance with few exceptions from the original version. Health responsibility (HR) and spiritual growth (SG) subscales of HPLP-II and physical and psychological health dimensions scores of SF-36 scores correlated significantly (r > 0.63, *p* < 0.001) ensuring strong concurrent validity.

**Conclusions:**

The Sinhala version of HPLP-II adapted by us is a tool with high reliability and validity.

## Background

Health promotion is a fundamental strategy in healthcare that implies changes in behavior and the adoption of patterns that promote good health in order to improve the quality of life (QOL) of people [1]. As defined by the World Health Organization (WHO) Ottawa Charter for Health Promotion (1986), health promotion is “the process of enabling people to increase control over and improve their health” [2].

Health professionals obligate to promote health at the individual, group, and community levels. To achieve the targets of health promotion activities, assessment of their existing practices on health promoting behaviors (HPB) and their strengths and weaknesses need to be identified [3]. HPB are major determinants of health and are used in disease prevention [4].

The Health Promoting Lifestyle Profile-II (HPLP-II) [5] is a revision of the HPLP developed by Walker et al. [6]. It measures health promoting lifestyles as HPB by focusing on self-initiated actions and perceptions that serve to maintain or enhance the level of wellness, self-actualization, and fulfillment of the individual. It is a 52-item questionnaire composed of six subscales including health responsibility (HR), nutrition (N), physical activity (PA), stress management (SM), interpersonal relations (IR), and spiritual growth (SG).

The English version of HPLP-II has shown high internal consistency and test-retest reliability [5]. It has been translated into different languages including Spanish, Japanese, Arabic, Taiwanese, Turkish and Portuguese [7–12] and its validity and reliability have been verified.

Menopause is one of the most significant events in women’s lives. The evaluation of lifestyle with HPB is important when considering certain conditions and diseases encountered by postmenopausal women (PMW). Health promotion is particularly important for PMW, since healthy behaviors such as regular exercises and stress management can effectively reduce the severity of health problems and discomforts associated with menopause.

Health promoting lifestyle is not constant and possibly varies depending on the context, culture and language [13]. That emphasizes the importance of cultural adaptation and test the validity and reliability of a specific instrument in the particular population.

The original HPLP-II has been widely used to measure healthy women’s HPBs in many societies [14, 15]. However, studies related to HPB in Sri Lanka are sparse and this could partly be due to the lack of a valid instrument to measure HPB. A reliable instrument for the measurement of healthy lifestyle is therefore a necessity for understanding and addressing the health promotion needs of the PMW in Sri Lanka. Therefore, this study attempted to evaluate the applicability of HPLP-II to measure the HPB of PMW conversant in the Sinhala language in Sri Lanka, with the cross cultural adaptation and evaluation of psychometric properties.

## Methods

### Study design, setting and participants

This validation study was conducted in the Bope-Poddala Medical Officer of Health (MOH) area, Galle District, in Southern Sri Lanka. The study participants in both cross cultural adaptation and psychometric validation were healthy community dwelling PMW who attained menopause naturally with intact uterus. Those with severe medical and surgical conditions, mental illnesses, disabilities and impairments of musculoskeletal system, endocrine disorders (diabetes, thyroid etc.), women on hormone replacement therapy (HRT) and illiterate women were excluded. Postmenopausal status was determined based on self-reported menstrual history using the classification of Stages of Reproductive Aging Workshop (STRAW) [16] which is the cessation of menstruation within the previous 12 months after last menstruation.

### Cross cultural adaptation of HPLP-II to Sinhala language

The standard guidelines described by Beaton et al. [17] were followed for the cross cultural adaptation of the Sinhala version of HPLP-II. Forward translation of the original English version of HPLP-II was done by two independent health professionals. Then the items were consolidated in to a single questionnaire. The synthesized version was back translated to English language by two another independent health professionals. Then a group of experts (a physician, a gynecologist, a physiologist, an anatomist, a nursing academic and, forward and backward translators) independently reviewed all the versions of HPLP-II and a pre-final Sinhala version was decided ensuring its content validity. The face validity of the pre-final version of HPLP-II was ensured by having a focus group discussion (FGD) with 10 PMW. Then it was pretested among 30 PMW selected from another geographical location. These two steps further ensure the clarity, understandability and naturalness of items [18].

### Administration of Sinhala version of HPLP-II

The newly adopted Sinhala version of HPLP-II and the previously validated Sinhala version of the Short Form 36 (SF-36) survey [19, 20] were self-administered among 245 PMW selected from the university training area to evaluate its psychometric properties. These women were selected using the multistage cluster sampling technique. Out of the 18 Public Health Midwife divisions (smallest community health unit) of the Bope-Poddala MOH area, three were selected randomly which were sub-urban areas. Houses and the female householders aged beyond 40 years were identified using the electoral registers of the respective areas obtained from the Grama Niladari Officer (local community administrative authority) of each division. Women who got odd numbers when the houses were arranged in a single list were invited for the study (excluding the women who had exclusion criteria) until the required sample size was achieved.

For the sample size calculation, five respondents in to one variable ratio was used [21]. Sample size was calculated by multiplying the number of variables in the instrument (52 items) by five in order to achieve the best sample (*n* = 260). After adding 10% to compensate with the non-respondents and incomplete questionnaires, the final sample size was calculated as 286. Even though we invited 286 women, only 272 women participated and only 245 PMW completed all the items in the questionnaires (response rate was 90%).

The HPLP-II asks respondents to indicate how often they adapt to HPB with 52 items (statements) listed under six subscales and answers are required to be given on a 4-point Likert scale (never, sometimes, often, and routinely). The Short Form Survey (SF-36) provides a subjective estimation of the individual’s functional state and QOL in two main dimensions (physical and psychological) [19].

The SF-36 survey was used in this study assuming that lifestyle has a significant relationship with QOL meaning that good lifestyle practices improve the QOL [22, 23].

HPLP-II was re-administered among a randomly selected subgroup of PMW (*n* = 105) 2 weeks after the first administration.

### Statistical analyses

The socio-demographic characteristics of the study participants in psychometric validation step are presented as frequencies (percentages) or mean (SD). The test-retest reliability was examined by intra-class correlation coefficient (ICC) comparing the overall HPLP-II scores in two consecutive evaluations. Factor analysis (FA) performed with principal component analysis (PCA) evaluated the structural validity of the Sinhala version of HPLP-II. The Kaiser-Meyer-Olkin (KMO), Bartlett’s test of Sphericity statistics and correlation matrix were observed [24]. The percentage of variance explained by each component and number of Eigen values over one (Kaiser-Guttman rule) were identified [24]. Concurrent validity was evaluated by observing the Pearson correlation coefficients [24] between the HR subscale score of HPLP-II and physical health dimension score of SF-36, and between the SG subscale score of HPLP-II and psychological health dimension score of SF-36 survey. Statistical analysis was performed with SPSS 20.0 version and *P* value < 0.05 was considered as statistically significant.

### Ethical considerations

Permission from the original authors was sought before commencement of the process and ethical clearance for the study was obtained from the Ethical Review Committee, Faculty of Medicine, University of Ruhuna. Written informed consent was obtained from each participant who wished to take part in the study before administering the questionnaires.

## Results

### Characteristics of the participants

Age of the subjects ranged from 47 to 64 years while age at menopause and time since menopause ranged from 40 to 59 years and 2 to 7 years, respectively (Table 1). Sociodemographic characteristics of the participated Sinhalese PMW and mean (SD) of subscale and overall HPB scores are presented in Table 1.

**Table 1:** Basic characteristics of the study participants (*n* = 245)

Characteristic	Sub category	Mean (SD) or Frequency (%)
***Sociodemographic Characteristics***		
Age (years)		55.9 (3.4)
Age at menopause (years)		50.4 (2.9)
time since menopause (years)		5.6 (1.9)
Employment status	Employed	59 (24.1)
	Non employed or retired	186 (75.9)
Civil status	Married	208 (84.8)
	Single	16 (6.5)
	Widowed	13 (5.3)
	Divorced	8 (3.2)
Living companion	With husband and children	134 (54.6)
	With Husband or Children	86 (35.1)
	Alone or living with others	25 (10.2)
Education status	Primary education	105 (42.8)
	Secondary education	71 (29.0)
	Upper secondary education, degree or diploma	69 (28.1)
Monthly income	< 10,000 LKR	90 (36.7)
	10,000–20,000 LKR	95 (38.8)
	20,000–50,000 LKR	50 (20.4)
	> 50,000 LKR	10 (4.1)
***HPB scores***		
Health Responsibility		17.10 (5.93)
Nutrition		19.10 (4.99)
Physical Activity		12.50 (4.35)
Stress Management		16.19 (4.01)
Spiritual Growth		20.42 (4.87)
Interpersonal Relations		20.23 (4.54)
Overall HPB score		105.64 (26.61)

LKR = Sri Lankan rupees (180LKR = 1USD), *HPB* = Health promoting behaviors

Living with others include; parents, siblings, friends or other relatives

### Psychometric properties of Sinhala version of HPLP-II

#### Internal consistency and reliability

Sinhala version of HPLP-II showed a high internal consistency with global Cronbach’s alpha of 0.98, and six subscales: HR 0.95, N 0.91, PA 0.91, IR 0.90, SM 0.88 and SG 0.91 respectively. The test-retest reliability measured with ICC was 0.98 (95% CI = 0.97–0.99).

#### Structural validity

FA using PCA was performed. The correlation matrix revealed the presence of many coefficients> 0.3 (data not shown) while the KMO value was 0.89, and Bartlett’s Test of Sphericity reached statistical significance (*p* < 0.001) supporting the factorability of the correlation matrix and indicating the adequacy of sample size. PCA revealed the presence of seven factors with Eigen value exceeding one, explaining cumulative variance of 80.65% with varimax rotation in the rotated component matrix (each factor one to seven represent variances of 53.73, 7.71, 5.42, 4.78, 3.42, 3.08 and 2.49%). All 52 items in the Sinhala version of HPLP-II were representative of a component as the coefficient loading of all items was ≥0.40.

Factor 1 included a combination of items in all six domains (17 items), Factor 2 included items in the SM, IR and SG subscales (7 items), Factor 3 included items in the SG, IR and HR subscales (7 items), Factor 4 included items in the PA, N and SM subscales (6 items), Factor 5 included items in the SM, SG, IR and HR subscales (6 items), Factor 6 included items in the IR, N, PA and HR (6 items), and Factor 7 included only three items that were in the N and PA subscales (data not shown in tables).

Factors one to seven extracted in this analysis, showed satisfactory internal consistency; (Cronbach’s alpha values) and item total correlations. The Cronbach’s alpha for item 1 to 7 were 0.97, 0.92, 0.93, 0.93, 0.87, 0.92 and 0.82 respectively. The item-total correlations for item 1 to 7 were 0.64–0.91, 0.65–0.88, 0.74–0.85, 0.52–0.77, 0.71–0.82, 0.71–0.82 and 0.68–0.68 respectively.

#### Concurrent validity

The Pearson correlation coefficient observed between the HR score of HPLP-II and physical health dimension score of SF- 36 and SG score of HPLP-II and psychological health dimension score of SF-36 were high that indicated strong concurrent validity of the HPLP-II (Table 2).

**Table 2:** Correlation between HPLP-II scores and SF-36 scores

HPLP-II	SF-36 survey	
	Physical health dimension	Psychological health dimension
HR score	0.75^a^	–
SG score	–	0.63^a^

^a^Correlation is significant at < 0.001 level

HPLP-II = health promoting lifestyle profile-II, SF-36 = short form 36 survey, HR = health responsibility, SG = spiritual growth

## Discussion

The current study examined the ability of HPLP-II in evaluating the HPB of Sinhala speaking PMW in Sri Lanka. We observed that HPLP-II adapted to the Sinhala language has satisfactory reliability and validity indicating that the instrument is suitable for this purpose.

The items in the questionnaire were culturally adapted without compromising the original meaning but additional explanations relevant to the Sri Lankan context, such as, types of PA and food items were provided. Focus group discussions and pre-test analysis confirmed that the two-way translation process was successful in developing the Sinhala version of the HPLP-II and respondents found it easy to understand and complete.

The internal consistency and the reliability were higher in the questionnaire indicating its stability and repeatability. Structural validity evaluation in FA with PCA extracted seven factors that were different from the original factor structure of HPLP-II. These seven factors however explained a high variance of 80.65%. Further, concurrent validity of the questionnaire was tested against the SF-36 questionnaire, assuming that PMW who adhere to HPB have higher overall QOL. This study showed similarities of reliability and validity with previous studies while the factor structure differs in several aspects.

Internal consistency measured with Cronbach’s alpha of the entire questionnaire and subscales were concordant and at times exceeded the values observed elsewhere including the Turkish version [12], Taiwanese version [11], Spanish version [7] and Portuguese version [10]. Test-retest reliability was higher when compared with the original study [5], Iranian version [25], Taiwanese version [11], Spanish version [7] and Portuguese version [10].

In previous analyses, six main factors have appeared in the Iranian version [25], Taiwanese version [11], Spanish version [7] and Portuguese version [10]. However, in the Turkish version, 14 factors (explaining 61% of the variance) were found but the Scree plot graphic showed five factors, explaining 41% of the variance [12].

The correlation between the HPLP-II and QOL score is higher than that was observed in the Taiwanese version [11] in concurrent validity evaluation. This is the only study focused the evaluation of concurrent validity of HPLP-II.

The separation of items in the factor extraction in the current study can be due to many reasons including culture difference in attitudes and practices. Western women may interpret concepts such as “PA” as regimens describing jogging, lifting weights or swimming, whereas in Sri Lanka, PA is more likely to describe the day-to-day routine work. Furthermore, application of Kaiser’s criterion when more than 50 variables are involved also lead to the extraction of too many factors [26]. However, we observed that extracted factors had higher internal consistencies indicating that this factor extraction is more meaningful to the Sinhala language.

The higher correlation observed between HPLP-II and SF-36 scores in the current study could partly be due to the reference tool used to assess the concurrent validity. We used the SF-36 survey and the Taiwan study had used WHO-BREEF questionnaire. The scarcity of studies to evaluate the concurrent validity would be due to the absence of a proper tool to evaluate the correspondence of HPB. However, it is obvious that HPB increase or decrease the QOL, and therefore, we did not see any limitation in our approach in evaluating the concurrent validity. Illiterate women were not included in the study since the questionnaire was self-administered and the large number of items in the questionnaire could have influenced the quality of data. These are the few limitations of the current study. This validated questionnaire would help to collect better quality data in existing HPB of individuals. It increases the credibility of data related to the health promotion in all the individuals fluent in Sinhala language. Health promotion activities could be initiated based on the findings of this validated questionnaire.

Since Sri Lanka has a multi-ethnic community it is important to adapt a Tamil version of HPLP-II to suit the Tamil speaking community. It is also important to use a shortened version of this tool in future studies. Furthermore, confirmatory FA should be focused in those future studies.

## Conclusion

This study demonstrated linguistic and cultural acceptance and satisfactory psychometric properties; reliability and validity in the Sinhala version of HPLP-II.

## Data Availability

The datasets used and/or analyzed during the current study are available from the corresponding author on reasonable request.

## Abbreviations

HPLP-II: Health Promoting Lifestyle Profile-II
HPB: Health Promoting Behaviors
PMW: Postmenopausal Women
SF-36: Short Form 36 survey
ICC: Intra Class Correlation
QOL: Quality of Life
HR: Health Responsibility
N: Nutrition
PA: Physical Activity
SM: Stress Management
IR: Interpersonal Relations
SG: Spiritual Growth
MOH: Medical Officer of Health
HRT: Hormone Replacement Therapy
STRAW: Stages of Reproductive Aging Workshop
FA: Factor Analysis
PCA: Principle Component Analysis
KMO: Kaiser-Meyer-Olkin
